# Decreased Risk of Stroke in Patients with Traumatic Brain Injury Receiving Acupuncture Treatment: A Population-Based Retrospective Cohort Study

**DOI:** 10.1371/journal.pone.0089208

**Published:** 2014-02-19

**Authors:** Chun-Chuan Shih, Yi-Ting Hsu, Hwang-Huei Wang, Ta-Liang Chen, Chin-Chuan Tsai, Hsin-Long Lane, Chun-Chieh Yeh, Fung-Chang Sung, Wen-Ta Chiu, Yih-Giun Cherng, Chien-Chang Liao

**Affiliations:** 1 School of Chinese Medicine for Post-Baccalaureate, I-Shou University, Kaohsiung, Taiwan; 2 Neuroscience Laboratory, Department of Neurology, China Medical University Hospital, Taichung, Taiwan; 3 Graduate Institute of Integrated Medicine, College of Chinese Medicine, China Medical University, Taichung, Taiwan; 4 Department of Anesthesiology, Taipei Medical University Hospital, Taipei, Taiwan; 5 Health Policy Research Centre, Taipei Medical University Hospital, Taipei, Taiwan; 6 School of Medicine, College of Medicine, Taipei Medical University, Taipei, Taiwan; 7 Graduate Institute of Clinical Medical Science, China Medical University, Taichung, Taiwan; 8 Department of Public Health, China Medical University, Taichung, Taiwan; 9 Graduate Institute of Injury Prevention and Control, Taipei Medical University, Taipei, Taiwan; 10 Department of Anesthesiology, Shuang Ho Hospital, Taipei Medical University, New Taipei City, Taiwan; 11 Management Office for Health Data, China Medical University Hospital, Taichung, Taiwan; University of South Florida, United States of America

## Abstract

**Background:**

Patients with traumatic brain injury (TBI) face increased risk of stroke. Whether acupuncture can help to protect TBI patients from stroke has not previously been studied.

**Methods:**

Taiwan's National Health Insurance Research Database was used to conduct a retrospective cohort study of 7409 TBI patients receiving acupuncture treatment and 29,636 propensity-score-matched TBI patients without acupuncture treatment in 2000–2008 as controls. Both TBI cohorts were followed until the end of 2010 and adjusted for immortal time to measure the incidence and adjusted hazard ratios (HRs) with 95% confidence intervals (CIs) of new-onset stroke in the multivariable Cox proportional hazard models.

**Results:**

TBI patients with acupuncture treatment (4.9 per 1000 person-years) had a lower incidence of stroke compared with those without acupuncture treatment (7.5 per 1000 person-years), with a HR of 0.59 (95% CI = 0.50–0.69) after adjustment for sociodemographics, coexisting medical conditions and medications. The association between acupuncture treatment and stroke risk was investigated by sex and age group (20–44, 45–64, and ≥65 years). The probability curve with log-rank test showed that TBI patients receiving acupuncture treatment had a lower probability of stroke than those without acupuncture treatment during the follow-up period (p<0.0001).

**Conclusion:**

Patients with TBI receiving acupuncture treatment show decreased risk of stroke compared with those without acupuncture treatment. However, this study was limited by lack of information regarding lifestyles, biochemical profiles, TBI severity, and acupuncture points used in treatments.

## Introduction

Traumatic brain injury (TBI) is a common cause of disability and death in every age group and both sexes worldwide [1]–[3]. Health problems after TBI include neurologic deficit, cognitive impairment, psychiatric illness, poor social functioning, and other significant adverse outcomes such as brain tumors and mortality [2], [4]–[7]. This burden of disease calls for further investigation to prevent and treat complications after TBI [8].

Stroke is the second leading cause of death worldwide and the leading cause of acquired disability in adults in most regions [9]. An international multicenter study has identified cardiac diseases, hypertension, diabetes, smoking, alcohol intake, unhealthy diet, abdominal obesity, lack of exercise, psychosocial stress and depression as risk factors associated with 90% of stroke risk [10]. Prevention is an important way to reduce stroke incidence and mortality.

An increased risk of stroke among individuals who survive TBI has been documented [11], [12]. Acupuncture is a traditional Chinese medicine (TCM) treatment that is commonly used in Taiwan [13]–[17]. Acupuncture has been found to improve cognition and sleep quality for patients with TBI [18], and our previous report found that patients with TBI who receive acupuncture treatment had reduced use of emergency care and hospitalization in the first year after injury [19]. Acupuncture has been documented as part of rehabilitation for patients with TBI [20]. However, whether acupuncture is effective in preventing stroke for patients with TBI is unknown. This study investigates the effectiveness of acupuncture in decreasing stroke risk among patients with TBI using multivariate and immortal time adjustment in a nationwide population-based cohort study.

## Methods

### Ethics Statement

Insurance reimbursement claims used in this study were from Taiwan's National Health Insurance Research Database, which is available for public access. This study was conducted in accordance with the Helsinki Declaration. To protect personal privacy, the electronic database was decoded with patient identifications scrambled for further public access for research. Although National Health Research Institutes regulations do not require informed consent due to decoded and scrambled patient identification, this study was approved by Taiwan's National Health Research Institutes.

### Study Design and Population

Taiwan's National Health Research Institutes set up the National Health Insurance Research Database to allow access to all medical claims for insured beneficiaries since 1996. With patient identification numbers scrambled, data files can be secured to protect patient privacy. Information available for this study included gender, birth date, disease codes, health care rendered, medicines prescribed, diagnoses for admissions and discharges, and medical institutions and physicians providing services. This database was described in detail in our previous studies [1]–[3], [19], [21]–[25].

From a longitudinal cohort population-based database of a randomly selected one million insured subjects in 2000, we identified persons aged ≥20 years old with newly diagnosed TBI who made visits for medical care in 2000–2008 as our eligible study patients. In order to confirm that all patients with TBI in our study were incident cases, only new-onset TBI cases were included in this study; people with previous medical records of TBI within five years before the index date were excluded. The diagnosis of TBI was validated in previous studies [1]–[3]. Overall, we identified 37,045 new-onset TBI survivors aged ≥20 years; 7409 of them had used at least two courses (one course including six consecutive treatments) of acupuncture after TBI. We compared TBI patients receiving at least two courses of acupuncture treatment with patients without acupuncture treatment. TBI patients with only one course of treatment were excluded from this study. Each TBI patient was either followed up from the index date until 31 December 2010 or was censored. The follow-up time, in person-years, was calculated for each TBI patient until the diagnosis of stroke or until being censored due to death, withdrawal from the insurance system or loss to follow-up. The non-acupuncture group included patients with TBI who did not have acupuncture treatment before the end-point of follow-up. The person-years of TBI patients with acupuncture were calculated from the beginning of receiving acupuncture treatment corrected by immortal time [26].

### Criteria and Definition

We defined TBI according to the *International Classification of Diseases, 9th Revision, Clinical Modification* (ICD-9-CM 800–805, 850–854) [1]–[3], [19]. The primary outcome as the incident event of stroke was defined as ICD-9-CM 430–438. Coexisting medical conditions – hypertension (ICD-9-CM 401–405), mental disorders (ICD-9-CM 290–319), diabetes (ICD-9-CM 250), ischemic heart disease (ICD-9-CM 410–414), hyperlipidemia (ICD-9-CM 272.0–272.4), migraine (ICD-9-CM 346), and epilepsy (ICD-9-CM 345) – were considered as confounding factors due to documented increased risk of stroke in patients with these coexisting medical conditions [10], [11], [27]. TCM physicians were defined as physicians licensed by Taiwan's Department of Health who practiced TCM in regulated clinics or hospitals. We calculated the density of TCM physicians (TCM physicians/10,000 persons) using the number of TCM physicians per 10,000 residents for each administrative unit. The first, second, and third tertiles were considered as areas with low, moderate, and high physician density.

We identified stroke-related medications such as anticoagulants, antiplatelet agents, and lipid-lowering agents as potential confounding factors in the association between acupuncture treatment and stroke. National Health Insurance-covered anticoagulant included warfarin, dabigatran, heparin, and enoxaparin. Antiplatelet agents included aspirin, dipyridamole, ticlopidine, cilostazol, clopidogrel, tirofiban, aggrenox, abciximab, and eptifibatide. Lipid-lowering agents included atorvastatin simvastatin, rosuvastatin, fluvastatin, lovastatin, pitavastatin, pravastatin, vytorin (ezetimibe with simvastatin), gemfibrozil, and fenofibrate.

The selection of acupuncture point was determined by TCM doctors who made clinical assessment in accordance with TCM principles, such as the GV26 (Shuigou) point at the junction of the upper and middle third of the philtrum. [28] Acupuncturists used commercially available, single-use, sterile, and disposable stainless steel needles [18]. After the needles placed manually, they were left in place for around 15 minutes. All of this group of doctors obey these standard TCM guidelines to perform acupuncture treatments in the licensed clinical settings.

### Statistical Analysis

To reduce confounding effects, we developed a non-parsimonious multivariable logistic regression model to estimate a propensity score for acupuncture treatment. Clinical significance guided the initial choice of covariates, which included age, sex, low-income status, density of TCM physicians, types of TBI, mental disorders, hypertension, diabetes mellitus, ischemic heart disease, hyperlipidemia, migraine, and epilepsy. We used a structured iterative approach to refine this logistic regression model to achieve balance of covariates within the matched pairs. We then matched (without replacement) patients who had acupuncture treatment to those who did not by using a greedy matching algorithm with a calliper width of 0·2 SD of the log odds of the propensity score. Nearest-neighbor algorithm was applied to construct matched pairs, assuming that the proportion of 0·95 to 1·0 is perfect.

The chi-square tests were used to analyze categorized data (age group, sex, low income, density of TCM physicians, types of TBI, mental disorders, hypertension, diabetes mellitus, ischemic heart disease, hyperlipidemia, migraine, epilepsy, anticoagulants, antiplatelet agents, lipid-lowering agents, and new-onset stroke events) between patients with TBI who had received acupuncture treatment and those who had not. The mean and standard deviation of age for TBI patients with and without acupuncture treatment was compared by *t*-tests. We performed the multivariable Cox proportional hazard model to analyze the adjusted hazard ratios (HRs) and 95% confidence intervals (CIs) of stroke associated with acupuncture treatment in patients with TBI. All analyses were performed using Statistical Analysis Software version 9.1 (SAS Institute Inc., Cary, North Carolina, USA). A two-sided probability value of <0.05 was considered significant.

## Results

After propensity-score matching procedure (**Table 1**) there was no significant difference in age, sex, low income, area with TCM physician density, types of TBI, mental disorders, hypertension, diabetes, ischemic heart disease, hyperlipidemia, migraine and epilepsy between TBI patients with and without acupuncture treatment. Patients with acupuncture treatment had higher proportions of using anticoagulants (2.3% vs. 1.7%, p = 0.0003), antiplatelet agents (28.7% vs. 22.3%, p<0.0001), and lipid-lowering agents (24.7% vs. 18.4%, p<0.0001) compared with those had no acupuncture treatment. TBI patients who underwent acupuncture treatment had a lower proportion of new-onset stroke events than those without acupuncture treatment (2.2% vs. 4.2%, p<0.0001).

**Table 1:** Baseline characteristics and stroke events for traumatic brain injury patients with and without acupuncture treatment.

	Acupuncture		*p*-value
	No (N = 29636)	Yes (N = 7409)	
Sex	n (%)	n (%)	1.00
Female	14056 (47.4)	3514 (45.4)	
Male	15580 (52.6)	3895 (52.6)	
Age, years			1.00
20–29	9068 (30.6)	2267 (30.6)	
30–39	5824 (19.7)	1456 (19.7)	
40–49	5544 (18.7)	1386 (18.7)	
50–59	3804 (12.8)	951 (12.8)	
60–69	2844 (9.6)	711 (9.6)	
70–79	2080 (7.0)	520 (7.0)	
≥80	472 (1.6)	118 (1.6)	
Mean±SD	42.6±17.1	42.5±16.9	0.52
Low income			1.00
No	29228 (98.6)	7307 (98.6)	
Yes	408 (1.4)	102 (1.4)	
Density of TCM physicians			1.00
Low	4352 (14.7)	1088 (14.7)	
Moderate	11744 (39.6)	2936 (39.6)	
High	13540 (45.7)	3385 (45.7)	
Type of TBI			1.00
Mild	12868 (43.4)	3217 (43.4)	
Moderate	5780 (19.5)	1445 (19.5)	
Severe	10988 (37.1)	2747 (37.1)	
Coexisting medical conditions			
Mental disorders	2708 (9.1)	677 (9.1)	1.00
Hypertension	3084 (10.4)	771 (10.4)	1.00
Diabetes mellitus	1324 (4.5)	331 (4.5)	1.00
Ischemic heart disease	608 (2.1)	152 (2.1)	1.00
Hyperlipidemia	452 (1.5)	113 (1.5)	1.00
Migraine	124 (0.4)	31 (0.4)	1.00
Epilepsy	60 (0.2)	15 (0.2)	1.00
Stroke-related medications			
Anticoagulants	493 (1.7)	169 (2.3)	0.0003
Antiplatelet agents	6617 (22.3)	2127 (28.7)	<0.0001
Lipid-lowering agents	5462 (18.4)	1826 (24.7)	<0.0001
New stroke events	1250 (4.2)	163 (2.2)	<0.0001

**Figure 1:**
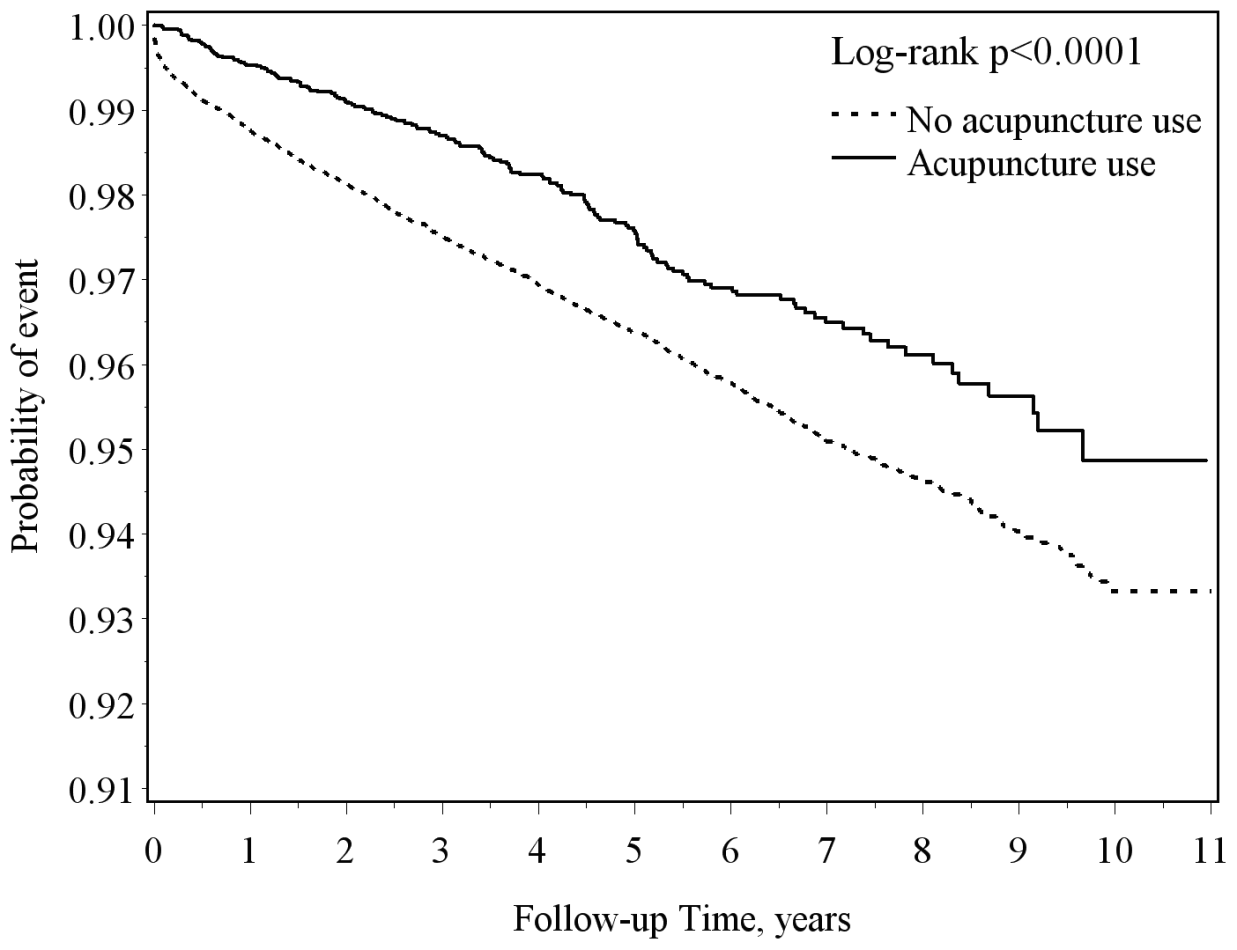
The stroke-free proportions estimated for TBI patients with and without acupuncture treatment using the Kaplan-Meier method.

During the follow-up period (**Table 2**), TBI patients with acupuncture treatment (4.9 per 1000 person-years) had a lower incidence of new-onset stroke than those without acupuncture treatment (7.5 per 1000 person-years), with a HR of 0.59 (95% CI = 0.50–0.69) after adjustment for age, gender, low income, TCM physician density, type of TBI, diabetes mellitus, hypertension, hyperlipidemia, mental disorder, ischemic heart disease, migraine, epilepsy, anticoagulants, antiplatelet agents, and lipid-lowering agents. Among patients with TBI, the decreased risk of new-onset stroke associated with acupuncture treatment showed no significant gender difference in (men, HR = 0.57, 95% CI = 0.46–0.71; women, HR = 0.62, 95% CI = 0.48–0.79). The age stratified results showed that the adjusted HR of stroke associated with acupuncture treatment for TBI patients was the lowest in the younger group aged 20–44 years. The further log-rank test (**Figure 1**) showed that TBI patients with acupuncture treatment had a lower probability of new-onset stroke events than those without acupuncture treatment (p<0.0001).

**Table 2:** Incidence, adjusted hazard ratios and confidence intervals of new-onset stroke for TBI patients with and without acupuncture treatment in the stratification of sex and age.

	Non-acupuncture				Acupuncture treatment					
	n	Events	Person-years	Incidence*	n	Events	Person-years	Incidence*	IRR (95% CI)	HR (95% CI)
Overall†	29636	1250	173682	7.5	7409	163	33071	4.9	0.68 (0.58–0.81)	0.59 (0.50–0.69)
Sex‡										
Female	14056	550	84939	6.5	3514	73	15890	4.6	0.71 (0.55–0.91)	0.62 (0.48–0.79)
Male	15580	700	88743	7.9	3895	90	17181	5.2	0.66 (0.53–0.83)	0.57 (0.46–0.71)
Age§										
20–44	17781	235	109647	2.1	4420	24	20054	1.2	0.56 (0.35–0.85)	0.46 (0.30–0.71)
45–64	7854	480	45206	10.6	1997	66	8953	7.4	0.69 (0.53–0.90)	0.64 (0.50–0.83)
≥65	4001	535	18829	28.4	992	73	4064	18.0	0.63 (0.49–0.81)	0.60 (0.47–0.76)

*Per 1000 person-years with calculated by correcting immortal time.

† Adjusted for age, gender, low income, density of TCM physicians, types of TBI, diabetes mellitus, hypertension, hyperlipidemia, mental disorders, ischemic heart disease, migraine, epilepsy, anticoagulants, antiplatelet agents, and lipid-lowering agents.

‡ Adjusted for all covariates in the full model except gender.

§ Adjusted for all covariates in the full model except age.

CI, confidence interval; HR, hazard ratio; IRR, incidence rate ratio; TBI, traumatic brain injury; TCM traditional Chinese medicine.

## Discussion

Using Taiwan's National Health Insurance Research Database, we conducted a retrospective cohort study with comprehensive design (matching procedure of propensity score) and showed significantly decreased risk of new-onset stroke events for patients with TBI who received acupuncture treatment. The present study is the first to report that acupuncture treatment was associated with reduced stroke risk for patients with TBI.

### Confounding Effects

Male, older age, low income and coexisting medical conditions are risk factors for TBI and post-TBI outcomes [1]–[3]. The use of TCM or acupuncture was associated with age, sex, low-income status and chronic diseases such as hypertension, mental disorders, diabetes, stroke, ischemic heart diseases, hyperlipidemia, migraine, and epilepsy. [13]–[17], [19]. To properly evaluate whether acupuncture treatment is associated with reduced stroke risk in TBI patients, we used propensity score to match the difference of age, sex, low income, and density of TCM physicians, mental disorders, hypertension, diabetes, ischemia heart disease, hyperlipidemia, migraine and epilepsy between TBI patients with and without acupuncture treatment. To accurately estimate risk of stroke after TBI for patients with and without acupuncture treatment, residual confounding effects were adjusted in the multivariable Cox proportional hazard models.

### Possible Explanations

Our previous study found that patients with TBI who received acupuncture treatment had less emergency care and hospitalization in the first year after injury compared with control [19]. In a small sample of patients with TBI, Zollman et al. proved that acupuncture improves cognition and perception of sleep or sleep quality [18]. A clinical trial showed the intervention of acupuncture combined with point-injection in TBI patients improved post-TBI aphasia, hemiplegia, and injuries of cranial nerves (including injuries of the facial, oculomotor and abducent nerves) [29]. This study found that TBI patients who had acupuncture had decreased risk of stroke.

We propose two possible explanations. First, acupuncture has biological benefits for TBI patients. Several studies show acupuncture's effectiveness in improving stroke patients' physical abilities [30], [31]. It has been shown that acupuncture is useful in lowering blood pressure [32], [33], reducing inflammatory mediators [34], and improving lipid profile [35], [36]. Acupuncture may also mediate anti-pain, anti-anxiety, and other therapeutic effects via intrinsic neural circuits that influence the affective and cognitive dimensions of pain [37]. Modulation of subcortical structures may also be an important mechanism by which acupuncture exerts complex multisystem effects [38]. This modulation and sympathy-vagal response may relate to acupuncture's analgesia and other potential therapeutic effects [39]. These findings implied that acupuncture might improve physical activity to reduce the risk of stroke. Second, patients with TBI who choose acupuncture treatment may have better knowledge, attitudes and practices regarding physical rehabilitation and disease prevention, which we believe could also contribute to reduce new-onset stroke event after TBI.

### Study Strengths

Among this study's strengths is its large sample, as it uses a representative sample of one million subjects from Taiwan's National Health Insurance Research Database. Second, our study design was a retrospective cohort; this provides more evidence than case-control or cross-sectional designs. Third, to eliminate the interference of sociodemographics and coexisting medical conditions between TBI patients with or without acupuncture treatment, we used propensity score matching procedure to select acupuncture treatment and non-treatment controls. To control residual confounding effects in the association between decreased risk of stroke after TBI and acupuncture treatment, we applied the multivariable Cox proportional hazard models to calculate adjusted HRs and 95% CIs of stroke associated with acupuncture treatment. Finally, immortal time in observational studies can bias the results in favor of the treatment group, but it is difficult to identify and avoid [27]. To reduce such bias, we calculated person-years to correct immortal time in the group with acupuncture treatment.

### Study Limitations

This study has several limitations. First, we used retrospective medical claims data from health insurance that lacked detailed patient information on lifestyle as well as physical, psychiatric, and laboratory examinations. Second, we used ICD-9-CM codes claimed by physicians for TBI without clarifying the severity of disease using means such as the Glasgow coma scale. Third, the data provided by insurance claims might underestimate the prevalence of TBI due to cases in which patients with very minor TBI might not seek medical treatment. In addition, the beneficial effects from acupuncture were somewhat different from individual acupuncture points [40]. Our study could not validate the actual acupuncture points used in treatment due to the limited information from the National Health Insurance Research Database. Finally, the mode of acupuncture treatment for patients with TBI varied with TCM physicians. We could not confirm every TCM physician performed the same procedures and acupuncture points for patients with TBI.

### Conclusions

From the results of this nationwide retrospective cohort study with matching procedure by propensity score, multivariable adjustment and immortal time correction, we suggested that TBI patients with acupuncture treatment had lower risk of new-onset stroke compared with TBI patients without acupuncture treatment. The association between acupuncture treatment and decreased risk of stroke among TBI patients existed in both sexes and among all age groups. However, further investigation is needed on specific acupuncture points to detail the mechanisms for such effects.
